# The flavonoid rutin protects the bumble bee *Bombus impatiens* against cognitive impairment by imidacloprid and fipronil

**DOI:** 10.1242/jeb.244526

**Published:** 2022-09-09

**Authors:** Andre J. Riveros, Wulfila Gronenberg

**Affiliations:** 1 Departamento de Biología, Facultad de Ciencias Naturales, Universidad del Rosario, Bogotá, Colombia; 2 Department of Neuroscience, School of Brain, Mind and Behavior, University of Arizona, Tucson, AZ 85721, USA

**Keywords:** Flavonol, Neonicotinoid, Bee decline, Pollinator

## Abstract

The ongoing decline of bee populations and its impact on food security demands integrating multiple strategies. Sublethal impairments associated with exposure to insecticides, affecting the individual and the colony levels, have led to insecticide moratoria and bans. However, legislation alone is not sufficient and remains a temporary solution to an evolving market of insecticides. Here, we asked whether bees can be prophylactically protected against sublethal cognitive effects of two major neurotoxic insecticides, imidacloprid and fipronil, with different mechanisms of action. We evaluated the protective effect of the prophylactic administration of the flavonoid rutin, a secondary plant metabolite, present in nectar and pollen, and known for its neuroprotective properties. Following controlled or *ad libitum* administration of rutin, foragers of the North American bumble bee *Bombus impatiens* received oral administration of the insecticides at sublethal realistic dosages. Learning acquisition, memory retention and decision speed were evaluated using olfactory absolute conditioning of the proboscis extension response. We show that the insecticides primarily impair acquisition but not retention or speed of the conditioned proboscis extension response. We further show that the administration of the flavonoid rutin successfully protects the bees against impairments produced by acute and chronic administration of insecticides. Our results suggest a new avenue for the protection of bees against sublethal cognitive effects of insecticides.

## Abstract (precis)

**Highlighted Article:** Prophylactically feeding bumble bees with rutin protects their learning and memory performance against oral exposure to insecticides with different mechanisms of action.

## INTRODUCTION

The decline of populations of managed and wild bees is a global concern because of their services provided through pollination (Bailes et al., 2015; Peixoto et al., 2022; Wood et al., 2020). Among the causes of the decline, insecticides have been the major culprit because of their lethal and sublethal effects. Sublethal impacts are particularly critical because of the subtle impairment of individuals, later translated into the deterioration of colony performance (Aliouane et al., 2009; Crall et al., 2018). For example, neuroactive insecticides alter neuronal activity (Dupuis et al., 2012; Palmer et al., 2013), lead to oxidative stress (Gregorc et al., 2018; Martelli et al., 2020), impair mitochondrial function (Moffat et al., 2015) and eventually lead to neurodegeneration (Peng and Yang, 2016). Consequently, bees exhibit impairments in motor control (Moffat et al., 2016; Williamson et al., 2014), sensory sensitivity (Démares et al., 2016, 2018; Eiri and Nieh, 2012; Kessler et al., 2015), learning and memory (Muth et al., 2019; Wright et al., 2015; Zhang and Nieh, 2015), and navigation abilities (Henry et al., 2012), even at low field-realistic doses.

Addressing the deceptively subtle effects on insect pollinators, the European Union (EU) banned and imposed restrictions on the use of major neuroactive insecticides, notably fipronil and the neonicotinoids imidacloprid, thiamethoxam and clothianidin (Butler, 2018; Gross, 2013; IPBES, 2016). Yet despite the legislation, concerns remain as insecticides are widely relied upon elsewhere, even within certain EU countries for non-flowering crops (Sonne and Alstrup, 2019) and alternative products, such as pyrethroids, may be prone to evolution of resistance, opening the door to pest outbreaks (Carreck, 2017; Gong et al., 2021; Hanson et al., 2017; Kathage et al., 2018; Lundin, 2021). Moreover, legislation alone may lead to an arms race between the development of new insecticides, the long-term scientific evaluation of sublethal effects, and the path toward new political action (Asher, 2018; Siviter and Muth, 2020; Siviter et al., 2018). Hence, future food security in the face of pollinator decline and potential pest outbreaks urgently calls for multilevel strategies.

Recently, bee nutrition has surged as a key factor in bee resilience against insecticides and other environmental stressors (Bernklau et al., 2019; Brodschneider and Crailsheim, 2010; Liao et al., 2017; Mao et al., 2013; Mitton et al., 2020; Negri et al., 2019). Specific nutrients such as caffeine, *p*-coumaric acid, quercetin, kaempferol and casein increase resilience against insecticides while enhancing pathogen tolerance and supporting the immune system (Bernklau et al., 2019; Folly et al., 2021; Liao et al., 2017; Mao et al., 2013; Mitton et al., 2020). Interestingly, some of these secondary metabolites from plants, such as flavonoids, are also intensely studied for their potential as remedies against human neurodegenerative diseases including Parkinson's and Alzheimer's disease (Maan et al., 2020; Maher, 2019; Pu et al., 2007; Sabogal-Guáqueta et al., 2015). Flavonoids are known for their antioxidant activity, mitochondrial stabilization, neural protection and regulation of multiple cellular pathways (Heim et al., 2002; Maan et al., 2020; Nkpaa and Onyeso, 2018; Punithavathi et al., 2010; Xu et al., 2019), and they are mass-produced as nutraceuticals and thus readily available. Might flavonoid supplements protect not just humans but also bees against neurocognitive impairments produced by insecticides?

Here, our approach was threefold. First, we evaluated whether the acute administration of a commercial form of imidacloprid, a neuroactive insecticide, impairs performance in learning and memory tasks in the North American bumble bee *Bombus impatiens*. We selected a commercial form of the insecticide because of the recognized effect of adjuvants, thus providing us with a more realistic, worst-case scenario for impairment and potential protection. Imidacloprid is a first-generation neonicotinoid that acts as a partial agonist of nicotinic acetylcholine receptors (nAChRs) and its sublethal effects in bees have been thoroughly assessed (IPBES, 2016). We selected bumble bees because they are relevant as managed and wild pollinators and are more sensitive to certain neuroactive insecticides relative to the European honey bee, *Apis mellifera* (Cresswell et al., 2012; IPBES, 2016; Moffat et al., 2016), thus being particularly well suited as an indicator for subtle deleterious effects*.* Sublethal effects of neuroactive insecticides on bumble bees include low reproduction (Whitehorn et al., 2012), altered thermoregulation (Crall et al., 2018; Potts et al., 2018) and cognitive impairment (Siviter et al., 2021a; Smith et al., 2020). Also, the consequences of exposure to insecticides extend beyond the individual, affecting colony function (Crall et al., 2018; Gill et al., 2012; Siviter et al., 2021b).

Most importantly, as part of our first goal, we investigated whether a controlled prophylactic administration of the flavonol rutin, a glycoside form of quercetin, would confer any protection against the impairment observed. Rutin and quercetin, as well as many other potentially beneficial flavonoids, are present in nectar and pollen across many plant species (De-Melo et al., 2018; Gullón et al., 2017; Kostić et al., 2019). The amount of rutin varies greatly among plant species and preparations as well as across different studies (Gullón et al., 2017). Establishing rutin's potential role as a protectant against insecticide effects on pollinators would support the relevance of enhancing plant diversity for conservation of bees and other pollinators and explain some of the negative effects of monocultures on bee health (Obregon et al., 2021). We selected rutin because it is associated with neural protection induced by multiple causes, including impact on AChRs (Pu et al., 2007; Richetti et al., 2011) and, in the presence of beta-glucosidases, can hydrolyze to quercetin, another flavonoid associated with the expression of detoxification enzymes (Zhang et al., 2012) and antagonism of apoptosis (Miao et al., 2021). Moreover, our previous results showed that partial protection of Africanized honey bees against imidacloprid was better after administration of rutin than after quercetin, probably as a result of a combined effect of rutin and its hydrolyzed form (L. M. García, J. J. Sutachan, C. F. Morantes-Ariza, V. Caicedo-Garzón, S. L. Albarracín and A.J.R., unpublished).

As a second goal, we set out to test whether the protection induced by rutin was independent of the mechanism of action of the insecticide and the controlled administration of the flavonoid. We selected an acute administration of fipronil, an insecticide primarily acting as an antagonist of gamma-aminobutyric acid (GABA) receptors. GABA is a neurotransmitter involved in motor control and information processing through inhibitory feedback within neural networks (El Hassani et al., 2005, 2009; Gauthier and Grünewald, 2012; Mustard et al., 2020). In this case, we also allowed *ad libitum* administration of rutin, which enabled us to test its innocuity. Finally, as a third goal, we aimed to test the limits of protection, by exposing the bees to *ad libitum* administration of the flavonoid followed by *ad libitum* administration of fipronil and imidacloprid.

Our results show that the acute and *ad libitum* administration of imidacloprid and fipronil impair learning acquisition and that the prophylactic administration of rutin leads to protection against the deleterious effects. We conclude that the prophylactic administration of rutin generally induces protection against learning and memory impairments induced by two insecticides with different mechanisms of action.

## MATERIALS AND METHODS

### Collection and maintenance of bees

We acquired three colonies of the bumble bee *Bombus impatiens* Cresson 1863 from Koppert Biological Systems Inc. (Howell, MI, USA). Colonies were maintained under laboratory conditions with an *ad libitum* supply of pollen (inside the nest) and 1 mol l^−1^ sucrose solution [external feeder attached to the supplied colony box through short (15 cm) transparent tubes]. For all experiments, we relied exclusively on forager bees collected from the external feeder. Bees from only a single colony were used for each of the three respective experiments described below to control for the potential variation among bees originating from different colonies.

### Dosage of insecticides

We determined the dose of insecticides based on the LD_50_ (the amount of an ingested substance that kills 50% of a test sample), the ‘realistic’ (field-reported) concentrations, the maximum volume imbibed by a forager, and the number of required training trials (to avoid satiation during training). Reports on LD_50_ concentrations of imidacloprid in bumble bees vary between 1 and 4 ng per bee (Riaño-Jiménez and Cure, 2016; Marletto et al., 2003). Based on this, we determined a low sublethal dose of 0.03 ng per bee, approximately 1/100 of the intermediate values reported for the LD_50_ in bumble bees. We have also previously established impairment of learning and memory in Africanized honey bees using such low doses (L. M. García, J. J. Sutachan, C. F. Morantes-Ariza, V. Caicedo-Garzón, S. L. Albarracín and A.J.R., unpublished). A bumble bee drinks about 150 µl of sugar water per foraging trip (Pattrick et al., 2020) or before satiation (A.J.R. and W.G., personal observations) and we aimed to provide the insecticide and conduct the experiments without satiating the bees. For training, we estimated 10 µl ingested per trial so that by the end of training the total amount consumed approximated 110 µl (after 11 trials, see below). Then, we determined a volume of 20 µl (minimum collected by a bumblebee; Pattrick et al., 2020) of a 5 nmol l^−1^ solution of imidacloprid (1.3 ppb) to administer 0.03 ng per bee. This concentration is within the range of the field-reported values, reaching up to 64.58 ng g^−1^ (65 ppb) nectar and 1.8 ng g^−1^ (1 ppb; Jiang et al., 2018) pollen. Hence, a bee receiving a controlled dose of imidacloprid (experiments 1 and 2) ingested in total 130 µl of liquid. Thus, satiation was not expected to affect performance.

For the dose of fipronil, the information is scarcer and whereas concentrations are reported, individual dosages (ng per bee) are not generally available for cognitive impairment. Thus, we followed our empirically determined dose of 1 ng per bee based on honey bees (L. M. García, V. Caicedo-Garzón and A.J.R., unpublished), which is below the reported LD_50_ of 4.2 ng per bee in honey bees (Pisa et al., 2015); previous results indicated behavioral impairment with this dose (L. M. García, V. Caicedo-Garzón and A.J.R., unpublished; El Hassani et al., 2005). We provided this dose in the form of 20 µl of a 0.11 μmol l^−1^ solution (experiment 2) unless the administration was *ad libitum* (experiment 3). Importantly, the concentration of 0.11 μmol l^−1^ (48 ppb) is within the reported range of fipronil in nectar (2.3–70 ppb; reviewed by Bonmatin et al., 2015).

### Training apparatus

The training apparatus has been previously described (Jernigan et al., 2014; Riveros and Gronenberg, 2009; Riveros et al., 2020). Briefly, the apparatus consists of 12 individual chambers, each hosting a bee restrained with a yoke in a plastic pipette tip. Each chamber is connected to a vacuum that cleans the air after odor stimulation. In front of the setup, there is a glass tube directed at the bees. The glass tube is connected to two currents of air originating from the same source. A first current is an ongoing stream of clean air whereas the second is controlled by valves and enables an overlapping flow of scented air used as a conditioning stimulus. Relying on two streams that originate from the same source guarantees that the overall flow is constant during a trial and that bees are not conditioned to the changes in pressure.

### Training procedure

#### General protocol

Bees were exposed to an odor for 10 s. The odor stimulus consisted of a piece of filter paper loaded with 5 μl of 1-nonanol (Alfa Aesar A12510) incorporated into the air current tube. Then, 7 s after the onset of odor presentation, the antennae were stimulated with 1.5 mol l^−1^ sucrose solution and, following a reflexive extension of the proboscis (proboscis extension response, PER), the bee was allowed to drink for 3 s while the odor current was still ongoing. We excluded the bees that did not respond to the sucrose solution with a PER. Each paired presentation was considered a training trial and each individual received 11 training trials with an average intertrial interval of 10 min. After training, all bees were fed 20 µl of 1 mol l^−1^ sucrose solution and maintained in plastic boxes with wet cotton until the retention test. Twenty-four hours after the last training trial, we presented the bees with the conditioned odor and recorded whether a bee exhibited a conditioned PER. Bees not exhibiting a conditioned PER were stimulated with 1 mol l^−1^ sucrose solution to test motivation. For each trial (acquisition and retention), we recorded whether a conditioned PER was exhibited and its latency in seconds using a metronome (2 Hz).

#### Experiment 1: protective effect of rutin against acute exposure to imidacloprid

We collected bees from colony 1 while on the feeder, anesthetized them on ice and yoke-restrained them in plastic pipettes. Bees were maintained in the pipettes for the entire duration of the experiment (5 days). During the first day, we randomly assigned each bee to one of two treatments: (i) 20 µl of 1 mol l^−1^ sucrose solution twice a day or (ii) 20 µl of 1 μmol l^−1^ rutin (600 ppb, 12 ng per bee; Sigma-Aldrich R5143) solution dissolved in 1 mol l^−1^ sucrose solution twice a day. This dosage of rutin (determined after L. M. García, J. J. Sutachan, C. F. Morantes-Ariza, V. Caicedo-Garzón, S. L. Albarracín and A.J.R., unpublished) is within or below the reported concentrations from the field, although there is enormous variation among plant species (1180 ppb in nectar reported by Guffa et al., 2017; Gullón et al., 2017). Bees received a total of six doses across three consecutive days. On the fourth day, we randomly assigned each bee to one of two treatments: (i) 20 µl of 1 mol l^−1^ sucrose solution or (ii) 20 µl of 5 nmol l^−1^ imidacloprid (1.3 ppb) solution (Prime Source LLC, Evansville, IN, USA) in 1 mol l^−1^ sucrose solution. Thus, each bee belonged to one of four treatments (Table 1): Control (sucrose for 3 days), Rut (rutin for 3 days), Imid (sucrose solution for 3 days and then imidacloprid before training), Rut+Imid (rutin for 3 days and then imidacloprid before training). Two hours after the administration of the solution, an experimenter blind to the treatments trained the bees using olfactory conditioning of the PER (see above). For the administration of insecticides in experiments 1–3, we stimulated the antennae with 1 mol l^−1^ sucrose solution to induce a PER and fed the insecticides directly to the tongue. Thus, the antennae were not contaminated with insecticides.

**Table 1.**
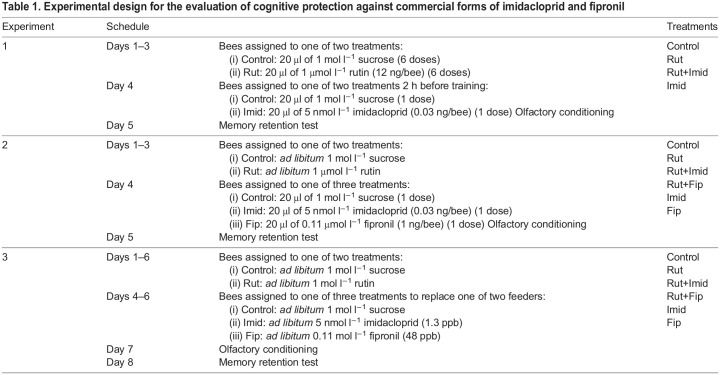
Experimental design for the evaluation of cognitive protection against commercial forms of imidacloprid and fipronil

#### Experiment 2: protective effect of *ad libitum* self-administration of rutin against acute exposure to imidacloprid and fipronil

We collected bees from colony 2 while on the feeder and randomly assigned each bee to one of two treatments: (i) 1 mol l^−1^ sucrose solution or (ii) 1 μmol l^−1^ rutin solution diluted in 1 mol l^−1^ sucrose solution. Bees were maintained in groups of 10 in plastic containers where they could freely walk and had *ad libitum* access to the feeding solutions (two vials with 1 ml were provided daily). On the night of the third day, we removed the feeders to starve the bees. On the fourth day, we ice anesthetized and yoke restrained the bees in plastic pipettes. Bees were maintained in the pipettes for the rest of the experiment (2 days). One hour after harnessing, we randomly assigned the bees to one of three treatments: (i) 20 μl of 1 mol l^−1^ sucrose solution, (ii) 20 μl of 5 nmol l^−1^ imidacloprid in 1 mol l^−1^ sucrose solution or (iii) 20 μl of 0.11 μmol l^−1^ fipronil (Taurus SC, Control Solutions Inc., Pasadena, TX, USA) in 1 mol l^−1^ sucrose solution. Thus, each bee belonged to one of six treatments (Table 1): Control (*ad libitum* sucrose for 3 days), Rut (*ad libitum* rutin for 3 days), Imid (*ad libitum* sucrose for 3 days and then acute imidacloprid before training), Rut+Imid (*ad libitum* rutin for 3 days and then acute imidacloprid before training), Fip (*ad libitum* sucrose for 3 days and then acute fipronil before training), Rut+Fip (*ad libitum* rutin for 3 days and then acute fipronil before training). Two hours after the administration of the solution, an experimenter blind to the treatments trained the bees using olfactory conditioning of the PER (see above).

#### Experiment 3: protective effect of *ad libitum* self-administration of rutin against *ad libitum* exposure to imidacloprid and fipronil

We collected bees from colony 3 while on the feeder and randomly assigned each bee to one of two treatments: (i) 1 mol l^−1^ sucrose solution or (ii) 1 µmol l^−1^ rutin solution diluted in 1 mol l^−1^ sucrose solution. Bees were maintained in groups of 10 bees in plastic containers and had *ad libitum* access to the feeding solutions as in experiment 2. Starting on the fourth day, we replaced one of the vials with one of three solutions: (i) 1 mol l^−1^ sucrose solution, (ii) 5 nmol l^−1^ imidacloprid in 1 mol l^−1^ sucrose solution or (iii) 0.11 μmol l^−1^ fipronil in 1 mol l^−1^ sucrose solution. Feeders were refilled daily for three additional days, providing a self-administered chronic exposure to insecticides. Thus, each bee belonged to one of six treatments (Table 1): Control (*ad libitum* sucrose for 6 days), Rut (*ad libitum* rutin for 3 days and then *ad libitum* sucrose and rutin for 3 days), Imid (*ad libitum* sucrose for 3 days and then *ad libitum* sucrose and imidacloprid for 3 days), Rut+Imid (*ad libitum* rutin for 3 days and then *ad libitum* rutin and imidacloprid for 3 days), Fip (*ad libitum* sucrose solution for 3 days and then *ad libitum* sucrose and fipronil for 3 days), Rut+Fip (*ad libitum* rutin for 3 days and then *ad libitum* rutin and fipronil for 3 days). On the night of the third additional day, we anesthetized and yoke restrained the bees in plastic pipettes. Bees were maintained in the pipettes for the rest of the experiment (2 days). The following day, an experimenter blind to the treatments trained the bees using olfactory conditioning of the PER.

#### Data analyses

For analyses, we included only bees exhibiting a PER to the sucrose solution across all training trials and the memory retention test. We calculated for each bee a learning score between 0 (no conditioned PER across the 10 trials) and 10 (conditioned PER across all 10 trails). The first training trial was not considered in the score and was used to determine that bees did not have a spontaneous PER to the conditioned stimulus. The scores within the planned comparisons were compared using a Wilcoxon test (one- or two-sided *P*-values depending upon predictions). Based on previous evidence, we predicted a decrease in performance of the bees exposed to insecticides and an improvement in bees prophylactically fed with rutin (relative to bees fed with insecticide and not fed with rutin). Of interest were the following comparisons: Control versus Fip/Imid to evaluate the effect of the insecticides, Control versus Rut to evaluate the innocuousness of rutin, Rut versus Rut+Imid/Rut+Fip, to test whether there was full protection, and Imid versus Rut+Imid and Fip versus Rut+Fip to test whether there was any significant protection. Differences in retention after 24 h were recorded as a nominal variable and compared relative to the last training trial using Fisher's exact test. At the population level, we constructed and analyzed learning curves using a generalized linear mixed model (GLMM). For the GLMM, we used a binomial structure with a Logit link function; also, we included Treatment (feeding schedule) and training Trial as fixed effects and Individual as a random effect. Latency of the conditioned PER was recorded as a continuous variable between 0.5 and 10 s. We averaged at least two conditioned responses (i.e. bees with only a single conditioned PER were not included in the latency analyses). Comparisons of conditioned PER latencies and body size (head width) were evaluated using an ANOVA if the distribution of data was normal (tested using a Shapiro–Wilk *W* test). In all cases, error due to multiple comparisons was controlled using the false discovery rate (FDR) one stage method (Pike, 2011; Verhoeven et al., 2005) and the corrected *P*-values (*q*-values) are presented. All the analyses were done using JMP v.16.1.0 (SAS Institute).

## RESULTS

### Experiment 1: protective effect of rutin against an acute exposure to imidacloprid

We collected and maintained 200 bees. Some bees were excluded because they escaped (*N*=8), died during the 3 day maintenance period before the exposure to pesticide (Control: *N*=5; Rut: *N*=2; Imid: *N*=2), did not exhibit a PER before training (Control: *N*=1; Rut: *N*=3; Imid: *N*=4; Rut+Imid: *N*=5) or at least once during training (Control: *N*=6; Rut: *N*=7; Imid: *N*=3; Rut+Imid=6), did not exhibit a PER during the retention test, or died during the 24 h period before the retention test (Control: *N*=4; Rut: *N*=4; Imid: *N*=1; Rut+Imid=3). Moreover, we conducted a screening of outliers for memory and learning score using Mahalanobis distances (0 bees excluded). Thus, we conducted our final tests using 136 bees distributed across four treatments: Control (*N*=34), Rut (*N*=35), Imid (*N*=36), Rut+Imid (*N*=31). Mean body size (head width) did not significantly differ across groups (mean±s.e.m. Control: 3.6±0.02 mm; Rut: 3.5±0.02 mm; Imid: 3.6±0.03 mm; Rut+Imid: 3.6±0.03 mm; ANOVA: *F*_3,132_=2.26, *P*=0.09).

**Fig. 1.**
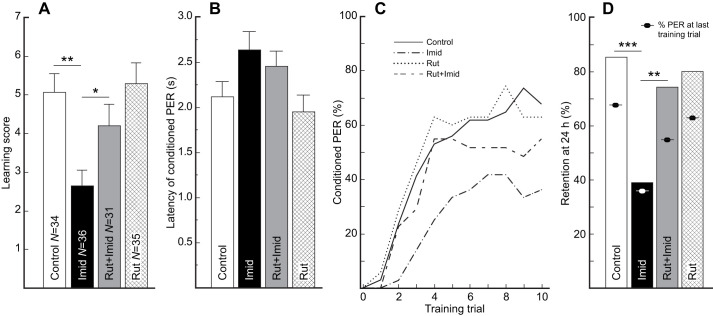
**Controlled prophylactic administration of rutin protects learning and memory against acute exposure to imidacloprid.** (A) Innocuous and protective effect against imidacloprid (Imid) of rutin (Rut) administration on learning. (B) Average latency of the conditioned proboscis extension response (PER) for bees that responded at least twice during training. (C) Acquisition curves for bees in all treatments. (D) Memory retention 28 h after exposure to imidacloprid and 40 h after the last administration of rutin. A, C and D include the same bees. Only statistically significant planned comparisons (see Materials and Methods) are indicated to facilitate visualization in A and D. Data are means±s.e.m. *P<0.05, **P<0.01, ***P<0.001.

Overall, we found that the administered compounds affected the level of performance of bees among treatments as indicated by the learning score (Kruskal–Wallis test: χ_3_^2^=15.81, *P*=0.0012). We found that the administration of imidacloprid significantly impaired the performance of bees. Relative to Control, the bees exposed to imidacloprid exhibited significantly lower learning scores (Control: 5.1±0.49; Imid: 2.6±0.42; Wilcoxon test: *Z*=−3.37, *P*=0.0016; Fig. 1A) yet no significant differences in PER latency (Control: 2.1±0.17 s; Imid: 2.6±0.21 s; *t*_105_=2.0, *P*=0.1; Fig. 1B). In contrast, the administration of rutin was innocuous, such that the bees in the Rut group exhibited learning scores (Rut: 5.3±0.55; Wilcoxon test: *Z*=0.40, *P*=0.69; Fig. 1A), and PER latencies (Rut: 1.9±0.19 s; *t*_105_=−0.67, *P*=0.51; Fig. 1B) that did not significantly differ from those of Control bees. Most importantly, the bees in the Rut+Imid group exhibited learning scores (Rut+Imid: 4.2±0.57; Fig. 1A) that were significantly higher than those of bees in the Imid group (Wilcoxon test: *Z*=2.0, *P*=0.046) but were not significantly different from those of Rut bees (Wilcoxon test: *Z*=1.41, *P*=0.21; Fig. 1A). Moreover, PER latencies of bees in the Rut+Imid group (2.44±0.17 s; Fig. 1B) were not significantly different from those of bees in the Rut group (Rut+Imid versus Rut, *t*_105_=−1.89, *P*=0.12) or in the Imid group (Rut+Imid versus Imid, *t*_105_=−0.65, *P*=0.35).

Like the analysis focused on individual scores, we found that acquisition at the population level was significantly affected by the training protocol and the feeding treatment (Fig. 1C). We observed an increase in the percentage of conditioned PER across training trials (Trial: GLMM: *F*=314.04, *P*<0.0001), and a significant variation in the level of PER across treatments (Treatment: GLMM: *F*=103.1, *P*=0.0018). We did not find a significant interaction between the treatment and the training trial (Treatment×Trial: GLMM: *F*=1.4, *P*=0.26), indicating that the overall pattern of acquisition across trials was not different. Thus, we conducted a new analysis on the reduced model. We found that bees were significantly affected by the training trial (Trial: GLMM: *F*=311.9, *P*<0.0001) and the treatment (Treatment: GLMM: *F*=5.78, *P*=0.001). After detailed analyses on our planned comparisons on the least square means, we found that the bees administered with imidacloprid (Imid group) exhibited significantly lower acquisition than bees receiving only sucrose (Control; one-sided *t*=−3.5, *P*=0.0003). In contrast, we did not find significant differences in learning acquisition between bees exposed to rutin (Rut group) or bees receiving only sucrose (Control; *t*=0.19, *P*=0.85). Most importantly, we found that the bees prophylactically fed with rutin and then exposed to the insecticide exhibited significantly higher levels of acquisition relative to bees exposed to imidacloprid (Imid group) (Rut+Imid; one-sided *t*=2.14, *P*=0.017). Similarly important, we did not find differences between the learning acquisition of bees exposed only to rutin (Rut group) and bees prophylactically fed with rutin and then exposed to imidacloprid (Rut+Imid; *t*=−1.46, *P*=0.15).

After 24 h, we found that the overall pattern of response was maintained relative to the last training trial, with bees in the Imid group exhibiting the lowest percentage of conditioned PER (Fig. 1D). Bees in the Imid group exhibited significantly lower memory retention than Control bees (adjusted Wald test: proportion difference=0.46, *P*=0.0001) and the odds of remembering were estimated to be 9.1 times higher (95% confidence interval, CI=2.8–29.1) for Control bees relative to Imid bees. In contrast, bees in the Rut group exhibited a level of memory retention that did not significantly differ from that of Control bees (adjusted Wald test: proportion difference=–0.05, *P*=0.590). Importantly, bees in the Rut+Imid group exhibited levels of memory retention that were significantly higher than those of Imid bees (adjusted Wald test: proportion difference=0.35, *P*=0.001) but did not significantly differ from those of Rut bees (adjusted Wald test: proportion difference=0.06, *P*=0.590). Also, the odds of remembering were estimated to be 4.5 times higher (95% CI=1.6–12.9) for Rut+Imid bees relative to Imid bees. However, retention itself was not generally affected by administration of the treatments as shown by the comparison, within each treatment, of the probability of exhibiting a conditioned PER during the last training trial and during the 24 h retention test (Fisher's exact test: Control: *P*=0.15; Imid: *P*=1.0; Rut+Imid: *P*=0.18; Rut: *P*=0.19; Fig. 1D).

### Experiment 2: protective effect of *ad libitum* self-administration of rutin against an acute exposure to imidacloprid and fipronil

We collected and maintained 223 bees. Some bees were excluded because they did not exhibit a PER before training (Control: *N*=5; Rut: *N*=7; Imid: *N*=2; Rut+Imid: *N*=5; Fip: *N*=2; Rut+Fip: *N*=7) or at least once during training (Control: *N*=2; Rut: *N*=0; Imid: *N*=2; Rut+Imid: *N*=0; Fip: *N*=0; Rut+Fip: *N*=0), did not exhibit a PER during the retention test (Control: *N*=0; Rut: *N*=4; Imid: *N*=2; Rut+Imid: *N*=3; Fip: *N*=2; Rut+Fip: *N*=3) or died during the 24 h period before the retention test (Control: *N*=0; Rut: *N*=0; Imid: *N*=1; Rut+Imid: *N*=0; Fip: *N*=0; Rut+Fip: *N*=0). We excluded 29 additional bees following a screening of outliers for memory and learning score using Mahalanobis distances. Thus, we conducted our final tests using 147 bees distributed across six treatments: Control (*N*=30), Rut (*N*=22), Imid (*N*=23), Rut+Imid (*N*=21), Fip (*N*=26), Rut+Fip (*N*=25). Mean body size (head width) was slightly larger in the Fip group but overall did not significantly differ across groups (mean±s.e.m. Control: 3.6±0.03 mm; Rut: 3.6±0.04 mm; Imid: 3.6±0.05 mm; Fip: 3.7±0.04 mm; Rut+Imid: 3.6±0.05 mm; Rut+Fip: 3.6±0.04 mm; ANOVA: *F*_5,141_=1.98, *P*=0.09).

**Fig. 2.**
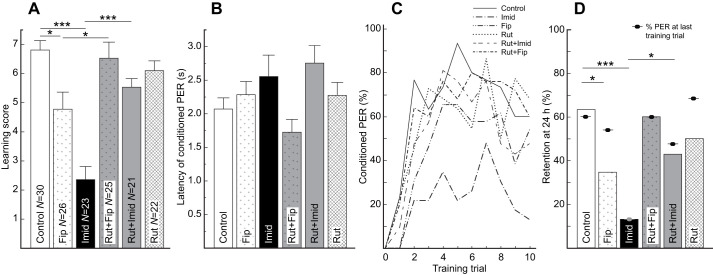
***Ad libitum* prophylactic administration of rutin protects learning and memory against impairment by acute exposure to imidacloprid and fipronil.** (A) Innocuous and fully protective effect against imidacloprid (Imid) and fipronil (Fip) of rutin (Rut) administration on learning. (B) Administration of insecticides did not significantly affect the latency of the conditioned PER. (C) Acquisition curves for bees in all treatments. (D) Innocuous and protective effect of rutin administration on memory retention 28 h after exposure to imidacloprid/fipronil and 40 h after the last administration of rutin. A, C and D include the same bees. Only statistically significant planned comparisons (see Materials and Methods) are indicated to facilitate visualization in A and D. Data are means±s.e.m. *P<0.05, ***P<0.001.

Overall, we found that the administered compounds affected the level of performance as indicated by the learning score (Kruskal–Wallis test: χ_5_^2^ =40.03, *P*<0.0001). We found that the administration of imidacloprid and fipronil significantly impaired the performance of bees. Relative to Controls, the bees exposed to imidacloprid and fipronil exhibited significantly lower learning scores (Control: 6.8±0.3; Imid: 2.4±0.46; Fip: 4.8±0.6; Wilcoxon test Control versus Imid: *Z*=−5.25, *P*=0002; Wilcoxon test Control versus Fip: *Z*=−2.5, *P*=0.014; Fig. 2A), but not significantly different PER latencies (Control: 2.1±0.2 s; Imid: 2.6±0.3 s; Fip: 2.3±0.2 s; Control versus Imid, *t*_126_=1.50, *P*=0.96; Control versus Fip, *t*_126_=0.73, *P*=0.96; Fig. 2B). In contrast, the bees in the Rut group exhibited learning scores (Rut: 6.1±0.35; Wilcoxon test Control versus Rut: *Z*=−1.61, *P*=0.15; Fig. 2A) and PER latencies (Rut: 2.3±0.2 s; Control versus Rut, *t*_126_=−0.71, *P*=0.96; Fig. 2B) that did not significantly differ from those of Control bees.

Remarkably, bees in the Rut+Imid and the Rut+Fip groups exhibited learning scores (Rut+Imid: 5.5±0.3; Rut+Fip: 6.5±0.6) that were significantly higher than those of bees in the Imid and Fip groups, respectively (Wilcoxon test for Rut+Imid versus Imid: *Z*=4.21, *P*=0.0002; Wilcoxon test for Rut+Fip versus Fip: *Z*=2.16, *P*=0.027; Fig. 2A) but were not significantly different from those of Rut bees (Wilcoxon test for Rut+Imid versus Rut: *Z*=0.98, *P*=0.33; Wilcoxon test for Rut+Fip versus Rut: *Z*=−1.18, *P*=0.28; Fig. 2A). Also, the PER latencies of bees in the Rut+Imid group (2.8±0.27 s) were not significantly different from those of bees in the Rut group (Rut+Imid versus Rut, *t*_126_=−1.55, *P*=0.21; Fig. 2B) or from bees in the Imid group (Rut+Imid versus Imid, *t*_126_=0.58, *P*=0.96; Fig. 2B). Finally, the PER latency of bees in the Rut+Fip group (1.7±0.2 s) was not significantly different from that of bees in the Rut group (Rut+Fip versus Rut, *t*_126_=1.80, *P*=0.96; Fig. 2B) or bees in the Fip group (Rut+Fip versus Fip, *t*_126_=−1.81, *P*=0.21; Fig. 2B).

As for analyses at the individual level, we found that, at the population level, bees in all groups were significantly affected by the training protocol and the feeding treatment (Fig. 2C). We observed an increase in the percentage of conditioned PER across training trials (Trial: GLMM: *F*=131.5, *P*<0.0001), but a significant variation in the level of PER across treatments (Treatment: GLMM: *F*=11.0, *P*<0.0001). We did not find a significant interaction between the treatment and the training trial (Treatment×Trial: GLMM: *F*=1.6, *P*=0.17), indicating that the overall patterns across trials were not different. Thus, we conducted a new analysis of the reduced model. We found that bees were significantly affected by the training trial (Trial: GLMM: *F*=135.9, *P*<0.0001) and the treatment (Treatment: GLMM: *F*=11.9, *P*<0.0001). After detailed analyses on our planned comparisons on the least squares means, we found that the bees administered with the insecticides (Imid and Fip groups) exhibited significantly lower acquisition than bees receiving only sucrose (Control; Imid versus Control: one-sided *t*=−6.91, *P*<0.0001; Fip versus Control: one-sided *t*=−3.21, *P*=0.0009). In contrast, we did not find significant differences in learning acquisition between bees exposed to rutin (Rut) or bees receiving only sucrose (Control; *t*=−1.14, *P*=0.26). Most importantly, we found that the bees prophylactically fed with rutin and then exposed to the insecticides exhibited significantly higher levels of acquisition relative to bees exposed to imidacloprid (Imid versus Rutin+Imid: one-sided *t*=4.77, *P*<0.0001) and fipronil (Fip versus Rut+Fip: one-sided *t*=2.76, *P*=0.0033) alone. Also, the bees prophylactically fed with rutin and then exposed to imidacloprid (Rut+Imid versus Rut: *t*=0.70, *P*=0.48) or fipronil (Rut+Fip versus Rut: *t*=−0.81, *P*=0.42) exhibited acquisition levels that were not different from those of bees fed only with rutin (Rut).

After 24 h, we found that the overall pattern of response was maintained relative to the last training trial, with bees in the Imid and Fip groups exhibiting the lowest percentages of conditioned PER (Fig. 2D). Bees in the Imid and the Fip groups exhibited significantly lower memory retention than Control bees (adjusted Wald test for Imid versus Control: proportion difference=0.52, *P*=0.0004; adjusted Wald test for Fip versus Control: proportion difference=−0.31, *P*=0.035) and the odds of remembering were estimated to be 12.7 times higher (95% CI=3.0–53.2) for Control bees relative to Imid bees and 3.6 times higher relative to Fip bees (95% CI=1.2–10.9). In contrast, bees in the Rut group exhibited a level of memory retention that did not significantly differ from that of Control bees (adjusted Wald test: proportion difference=−0.16, *P*=0.39).

Importantly, bees in the Rut+Imid group exhibited levels of memory retention that were significantly higher than those of Imid bees (adjusted Wald test: proportion difference=0.30, *P*=0.035; Fig. 2D) but did not significantly differ from those of Rut bees (adjusted Wald test: proportion difference=0.07, *P*=0.65; Fig. 2D). The odds of remembering were estimated to be 5.0 times higher (95% CI=1.12–22.2) for Rut+Imid bees relative to Imid bees. In the case of the protection against fipronil, the bees in the Rut+Fip group exhibited levels of memory retention (62.5%) that were clearly higher than those of the bees in the Fip group (37.5%; Fig. 2D), yet the difference was not statistically significant after the one-stage FDR correction (adjusted Wald test: proportion difference=0.25, *P*=0.06; a two-stage FDR rendered *P*=0.038). However, the levels of memory retention of Rut+Fip bees did not significantly differ from those of Rut bees (adjusted Wald test: proportion difference=0.1, *P*=0.59), suggesting an intermediate level of memory protection.

However, retention itself was not generally affected by administration of the treatments, as shown by the comparison, within each treatment, of the probability of exhibiting a conditioned PER during the last training trial and during the 24 h retention test (Fisher's exact test: Control: *P*=1.0; Imid: *P*=1.0; Rut+Imid: *P*=1.0; Fip: *P*=0.26; Rut+Fip: *P*=1.0; Rut: *P*=0.36; Fig. 2D).

### Experiment 3: protective effect of *ad libitum* self-administration of rutin against chronic exposure to imidacloprid and fipronil

We collected and maintained 360 bees. During the maintenance period some of the bees died (Control: *N*=2; Rut: *N*=4; Imid: *N*=3; Fip: *N*=2; Rut+Imid: *N*=0; Rut+Fip: *N*=5). Because of constraints on time for yoke restraining, training and maintenance, we could only prepare 192 bees for evaluation. Before training, some bees were excluded because they did not exhibiting a PER (Control: *N*=4; Rut: *N*=1; Fip: *N*=2; Imid: *N*=5; Rut+Imid: *N*=3; Rut+Fip: *N*=3). After training, bees were excluded because they did not exhibit a PER during the retention test (Control: *N*=0; Rut: *N*=0; Fip: *N*=3; Imid: *N*=1; Rut+Imid: *N*=0; Rut+Fip: *N*=1) or died during the 24 h period before the retention test (Control: *N*=1; Rut: *N*=2; Fip: *N*=1; Imid: *N*=0; Rut+Imid: *N*=2; Rut+Fip: *N*=2). We excluded 30 additional bees following a screening of outliers for memory and learning score using Mahalanobis distances. Thus, we conducted our final tests using 131 bees distributed across six treatments: Control (*N*=24), Rut (*N*=24), Imid (*N*=15), Rut+Imid (*N*=24), Fip (*N*=25), Rut+Fip (*N*=19). Mean body size (head width) did not significantly differ across groups (mean±s.e.m. Control: 3.5±0.04 mm; Rut: 3.5±0.04 mm; Imid: 3.5±0.04 mm; Fip: 3.5±0.05 mm; Rut+Imid: 3.5±0.04 mm; Rut+Fip: 3.5±0.04 mm; ANOVA: *F*_5,125_=0.384, *P*=0.859).

We found that bees generally consumed all the compounds provided, but consumption varied across the compounds added to the sucrose solution. Sucrose solution consumption was highest in Control bees but similar between bees also consuming insecticide (Control: 195.5±3.5 μl; Fip: 150.5±8.5 μl; Imid: 155.3±10 μl). Similarly, consumption of rutin was estimated to be higher in Rut bees but similar in bees exposed to rutin and insecticide (Rut: 192.1±7.7 μl; Rut+Fip: 162.6±8.8 μl; Rut+Imid: 151.9±9.6 μl). Finally, estimated volumes of ingested insecticide were similar across treatments including the same chemical (Fip: 93.7±5.9 μl; Rut+Fip: 97.3±5.7 μl; Imid: 103.5±3.6 μl; Rut+Imid: 88.6±4.1 μl). These estimated ingested volumes clearly suggest higher dosages per bee (Imid: 0.13 ng per bee; Rut+Imid: 0.11 ng per bee; Fip: 4.5 ng per bee; Rut+Fip: 4.7 ng per bee) compared with our treatments where the dosage was controlled (experiments 1 and 2).

**Fig. 3.**
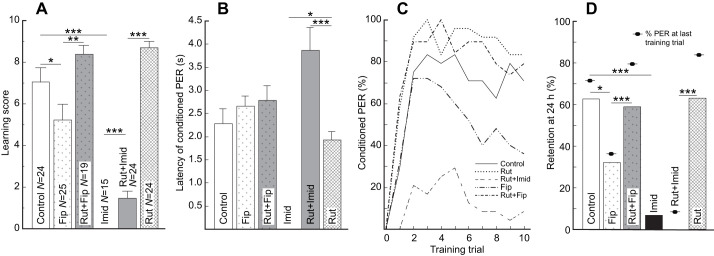
***Ad libitum* prophylactic administration of rutin protects learning and memory against impairment by chronic exposure to fipronil but not imidacloprid.** (A) Innocuous and protective effect against imidacloprid (Imid) and fipronil (Fip) of rutin (Rut). (B) Effect of feeding treatments on the latency of the conditioned PER of bees that responded at least twice. (C) Acquisition curves for bees in all treatments. (D) Effect of rutin administration on memory retention 40 h after the last administration of rutin. A, C and D include the same bees. Only statistically significant planned comparisons (see Materials and Methods) are indicated to facilitate visualization. Data are means±s.e.m. *P<0.05, **P<0.01, ***P<0.001.

Overall, we found that the administered compounds affected the level of performance, as indicated by the learning scores (Kruskal–Wallis test: χ_5_^2^=75.89, *P*<0.0001). We found that *ad libitum* administration of imidacloprid and fipronil impaired the performance of bees. Relative to Controls, the bees exposed to imidacloprid (Imid group) and fipronil (Fip group) exhibited significantly lower learning scores, although the effect was barely significant in the case of fipronil after FDR correction (Control: 7.0±0.7; Imid: 0.0±0.0; Fip=5.2±0.8 Wilcoxon test Control versus Imid: *Z*=−5.37, *P*=0.0003; Wilcoxon test Control versus Fip: *Z*=−1.78, *P*=0.05; Fig. 3A) while PER latencies did not differ significantly (Control: 2.3±0.3 s, *N*=20; Fip=2.6±0.2 s, *N*=18; Control versus Fip, *t*_84_=0.95, *P*=0.44). In contrast, the administration of rutin was innocuous such that the bees in the Rut group exhibited learning scores (Rut: 8.8±0.3; Wilcoxon test Rut versus Control: *Z*=1.45, *P*=0.175) and PER latency (Rut: 1.9±0.2 s, *N*=24; Control versus Rut, *t*_84_=−1.0, *P*=0.44) that did not significantly differ from those of Control bees.

Remarkably, bees in the Rut+Imid and the Rut+Fip groups exhibited learning scores (Rut+Imid: 1.3±0.3; Rut+Fip: 8.4±0.4) that were significantly higher than those of bees in the Imid and Fip groups, respectively (Wilcoxon-test for Rut+Imid versus Imid: *Z*=3.35, *P*=0.0009; Wilcoxon-test for Rut+Fip versus Fip: *Z*=2.69, *P*=0.007; Fig. 3A). However, bees in the Rut+Imid group exhibited learning scores that were significantly lower (Wilcoxon test for Rut+Imid versus Rut: *Z*=5.97, *P*=0.0003; Fig. 3A) and PER latencies that were significantly longer than those of bees in the Rut group (3.9±0.5 s, *N*=9; Rut+Imid versus Rut, *t*_84_=−4.17, *P*=0.0005; Fig. 3B). In contrast, bees in the Rut+Fip group exhibited learning scores that were not significantly different from those of bees in the Rut group (Wilcoxon test for Rut+Fip versus Rut: *Z*=0.43, *P*=0.67; Fig. 3A) and PER latencies that barely significantly differed from those of Rut bees after FDR correction (Rut+Fip: 2.8±0.3 s, *N*=18; Rut+Fip versus Rut, *t*_84_=−2.31, *P*=0.05; Fig. 3B). Also, bees in the Rut+Fip group exhibited PER latencies that were not significantly different from those of bees in the Fip group (Fip: 2.6±0.2 s; Rut+Fip versus Fip, *t*_84_=0.32, *P*=0.74; Fig. 3B).

When analyzing the population through the acquisition curves, we excluded bees in the Imid group as none of them exhibited a conditioned PER (Fig. 3C). We found that bees were affected by the training protocol and the feeding treatment (Fig. 3C; GLMM: Trial: *F*=61.3, *P*<0.0001; Treatment: *F*=21.7, *P*<0.0001). Importantly, we found a significant interaction between the feeding schedule and training trial (Treatment×Trial), indicating that the rate of acquisition was different between bees exposed to different treatments (GLMM: Trial×Treatment: *F*=9.9, *P*<0.0001). This significant effect was associated with the pattern of response of bees exposed to fipronil (determined after comparison of all GLMM pairs, not shown). In fact, a more detailed analysis showed that bees exposed to fipronil exhibited significantly lower acquisition than bees in the Control group (Fip versus Control: one-sided *t*=−1.95, *P*=0.027; Fig. 3C), with a decrease in the percentage of conditioned PER after the third trial. In contrast, bees fed with rutin exhibited significantly higher levels of acquisition than bees in the Control group (Rut versus Control: *t*=2.2, *P*=0.027; Fig. 3C). Also, bees prophylactically fed with rutin and then exposed to fipronil (Rut+Fip group) exhibited higher acquisition than bees exposed to fipronil (Fip versus Rut+Fip: one-sided *t*=3.30, *P*=0.0007; Fig. 3C). Moreover, bees fed prophylactically with rutin and then exposed to fipronil (Rut+Fip group) exhibited levels of acquisition that did not significantly differ from those of bees fed with rutin (Rut versus Rut+Fip: *t*=0.67, *P*=0.5). In contrast, bees prophylactically fed with rutin and then exposed to imidacloprid (Rut+Imid group) exhibited levels of acquisition that were significantly lower than those of bees fed with rutin alone (Rut+Imid versus Rut: *t*=8.37, *P*<0.0001).

We found that the overall pattern of response after 24 h was maintained relative to the last training trial, with bees in the Imid and Fip groups exhibiting the lowest percentage of conditioned PER (Fig. 3D). Bees in the Imid and the Fip groups exhibited significantly lower memory retention than Control bees (adjusted Wald test for Imid versus Control: proportion difference=0.55, *P*=0.0002; adjusted Wald test for Fip versus Control: proportion difference=0.31, *P*=0.036) and the odds of remembering were estimated to be 23.3 times higher (95% CI=2.6–208.6) for Control bees relative to Imid bees and 3.5 times higher relative to Fip bees (95% CI=1.1–11.5). In contrast, bees in the Rut group exhibited a level of memory retention that did not significantly differ from that of Control bees (adjusted Wald test: proportion difference=0.0, *P*=1.0). Interestingly, bees in the Rut+Imid group exhibited levels of memory retention that were not significantly different from those of Imid bees (adjusted Wald test: proportion difference=−0.07, *P*=0.25) but were significantly lower than those of Rut bees (adjusted Wald test: proportion difference=−0.62, *P*=0.0002). In the case of the protection against fipronil, the bees in the Rut+Fip group exhibited levels of memory retention that were significantly higher than those of bees in the Fip group (adjusted Wald test: proportion difference=0.31, *P*=0.036). The odds of remembering were estimated to be 3.6 times higher (95% CI=1.03–12.8) for Rut+Fip bees relative to Fip bees. Also, the levels of memory retention of Rut+Fip bees did not significantly differ from those of Rut bees (adjusted Wald test: proportion difference=0.007, *P*=1.0), suggesting a full level of protection. However, retention itself was not generally affected by administration of the treatments as shown by the comparison of the probability of exhibiting a conditioned PER during the last training trial and during the 24 h retention test (Fisher's exact test: Control: *P*=0.76; Imid: *P*=1.0; Rut+Imid: *P*=0.49; Fip: *P*=1.0; Rut+Fip: *P*=0.48; Rut: *P*=0.19).

## DISCUSSION

The negative ecological and economic impact of pesticides on pollinators, such as bees, calls for urgent and multidimensional approaches. For decades, evidence has highlighted the role of nutrition in the overall health of bees. Here, we tested whether rutin, a plant secondary metabolite, may protect against sublethal impairments induced by different schedules of oral administration of two neuroactive pesticides. Our results demonstrate that oral administration of both pesticides impairs primarily learning and, under some conditions, memory retention and decision speed. Most importantly, prophylactic administration of rutin, under well-defined doses as well as under *ad libitum* conditions, led to the development of protection against the pesticide effects. However, such protection was not observed in one case, when imidacloprid was provided *ad libitum* and led to a total depression of learning acquisition. Interestingly, memory retention was not affected, although a significantly lower performance was observed at 24 h. In contrast, bees prophylactically fed with rutin exhibited fully normal memory retention after administration of the insecticide, demonstrating protection. Thus, our results highlight three key aspects: sublethal impairment after exposure to imidacloprid and fipronil, the innocuous effect of rutin administration, and the protection provided by prophylactic administration of rutin against the pesticides.

First, exposure to imidacloprid and fipronil generally resulted in reduced learning performance as inferred from learning scores and acquisition curves (Figs 1A, 2A, 3A), but not to longer PER latencies (Figs 1B and 2B; but see Fig. 3B). Moreover, in most cases, bees exposed to the insecticide exhibited lower memory retention (but see below). These effects correspond with well-known impairments derived from multilevel effects by the insecticides and their metabolites. In our case, one of the insecticides, imidacloprid, is known to overstimulate the excitatory cholinergic pathway supporting olfactory learning, whereas fipronil suppresses the inhibitory GABAergic pathway. Eventually, the long-term action of insecticides and their metabolites may lead to neuronal degeneration, presumably as a consequence of mitochondrial instability inducing apoptosis (Peng and Yang, 2016; Wu et al., 2015). These long-term effects may help explain the sustained low performance observed, for example, 28 h after the administration of imidacloprid (Fig. 1D). However, low performance during retention tests may result from low learning (impaired acquisition) or impaired storage itself. In our case, the levels of memory retention were generally not significantly different from the performance observed during the last training trial, suggesting a long-term impact of the impairment in acquisition but not necessarily of retention. Nevertheless, the performance of bees administered with imidacloprid or fipronil was always low during the 24 h test, which argues for a long-term cognitive impairment. Thus, together, our results agree with the impairment observed in previous accounts following the administration of imidacloprid and fipronil, yet, suggest a larger variation in the response of the processes associated with memory storage and the speed of information processing. For example, fipronil led to apparent rapid acquisition followed by a decrease in the conditioned PER. Interestingly, the impairment was more severe with the administration of imidacloprid compared with fipronil, an effect probably due to the prevalence of ACh as an excitatory transmitter in the olfactory pathway during absolute conditioning (Grünewald, 2012, and references therein). As GABA acts as an inhibitory transmitter and contributes to odor discrimination (Wilson and Laurent, 2005), the impact of fipronil might be more significant during differential learning tasks as compared with our absolute conditioning protocol.

Second, and in contrast to the effects of the neuroactive insecticides, the prophylactic administration of rutin was innocuous. This result is not surprising given the extensive presence of rutin in nectar and pollen and the low dosages used here. Importantly, rutin has been found not to act as attractant or deterrent of bees and, unlike alkaloids and other allelochemicals, appears not to be toxic (Detzel and Wink, 1993). In our case, even under *ad libitum* administration of rutin we did not observe any negative effects on performance, although the individual amount ingested could not be quantified because of having multiple bees sharing a feeder. However, separate accounts from our group suggest that bees fed with rutin exhibit enhanced cognitive performance relative to Control bees and the learning curve of bees receiving *ad libitum* rutin was significantly higher. Finally, we highlight the fact that using very low concentrations of rutin allowed the dilution of the flavonoid in water. Rutin, like other flavonoids, is typically dissolved in dimethyl sulfoxide (DMSO) during bioassays, which greatly enhances its solubility. In honey bees, acute administration of DMSO has not been shown to cause impairments (Guez et al., 2005), yet its impact during extended schedules of exposure is unknown. Nevertheless, DMSO appears to exhibit significant levels of cell toxicity even at low concentrations (Galvao et al., 2014; Kim and Lee, 2021; Milchreit et al., 2016; Verheijen et al., 2019), making it an undesirable solvent for supplements that may require regular administration.

Third, we found that the performance of bees fed prophylactically with rutin and then exposed to the pesticides was significantly better than that of bees administered with the insecticides (Figs 1A, 2A and 3A). In all cases, the performance of ‘protected’ bees administered with fipronil was indistinguishable from that of bees fed only with rutin (Figs 2A and 3A). In the case of imidacloprid-treated bees, we observed full protection in two cases; however, when the bees were allowed *ad libitum* access to the insecticide, the ‘protection’ was low and partial. Nevertheless, this last scenario occurred when *ad libitum* imidacloprid appeared to fully impair the acquisition of any information (Fig. 3A), thus highlighting the sensitivity of bumble bees even at sublethal levels. These results reflect the subtler, yet significant, impact of fipronil relative to imidacloprid and the stronger deficiency derived from forcing the *ad libitum* self-administration of imidacloprid for 3 days (Moffat et al., 2016). This is particularly critical as imidacloprid induces a preference for neonicotinoid-laced sucrose solutions in bumble bees (Kessler et al., 2015). Furthermore, it highlights the variation in effects induced by different pesticides, previously reported even among related neonicotinoids (Moffat et al., 2016). However, direct comparisons are challenging because of the differences in the respective dosages used and the less documented toxicity of fipronil (presented as concentrations and not as dosage per bee).

The overall protection induced by the administration of rutin points to three elements of relevance. First, we observed protection for two neuroactive pesticides featuring distinct toxicodynamics. This implies a protection by rutin involving common targets of activity by imidacloprid and fipronil, such as the stability of mitochondrial function (Nicodemo et al., 2014; Powner et al., 2016) and the activation of detoxification mechanisms (Mao et al., 2011, 2013; Zhang et al., 2012). Importantly, rutin can be metabolized, enhancing its antioxidant and detoxification effects (De Araújo et al., 2013). Moreover, aiming to evaluate a more realistic scenario, we relied on commercial formulations of the pesticides; therefore, these results imply a protection against the adjuvants, which probably varied between pesticides and are broadly known to contribute to the observed impairments in bees (Mullin, 2015). Second, the protective effects seem to extend more than 36 h after the last dose of rutin. This is reflected by the normal performance of bees during the memory test, when the insecticide-treated bees had not recovered. Lastly, the latency of the PER of the protected bees tended to be longer than that of the bees in the rutin group (Fig. 3B), suggesting that rutin may improve the accuracy of the responses through effects on the speed of the decisions. Thus, together our results suggest that bumble bees can be protected against the sublethal cognitive impairment induced by commercial forms of two major pesticides acting through different mechanisms. Although the mitochondria appear to be a common cornerstone targeted by both the insecticides, in the absence of evidence on mitochondrial structure or function, our results do not allow us to confirm that the protection acts at this level.

Crop productivity is central to global issues associated with food security. Strategies such as the use of neurotoxic pesticides and establishing monocultures play a central role in maintaining productivity by decreasing damage through pests and enhancing crop yield of key products. Thus, it becomes crucial to develop and implement multiple strategies that secure food production while protecting non-target species, such as bees. Our approach of using a secondary metabolite of plants present in pollen and nectar introduces an alternative that protects the cognition of the bees at realistic levels of pesticide exposure. Our results suggest a direct use for managed species through specific supplementation; however, they also encourage higher plant diversity (for example through flowering species planting programs) to allow and enrich nutrition for wild bees and other pollinators (St Clair et al., 2020). Our results further call for evaluations in semi-field and field conditions, particularly addressing the issue of the population decline faced by managed pollinators. Considering the broad range of physiological effects of rutin (Heim et al., 2002; Nkpaa and Onyeso, 2018), one might expect rutin to positively affect other aspects of bee health in addition to protecting the nervous system. This study might also encourage future research into potential pollinator protective effects of other related secondary plant metabolites with established or assumed health effects for humans.
